# Real-time acoustic monitoring with telemetry to mitigate potential effects of seismic survey sounds on marine mammals: a case study offshore Sakhalin Island

**DOI:** 10.1007/s10661-022-10019-6

**Published:** 2022-10-18

**Authors:** Alexander N. Rutenko, Mikhail M. Zykov, Vladimir A. Gritsenko, Mikhail Yu. Fershalov, Michael R. Jenkerson, Roberto Racca, Vladimir E. Nechayuk

**Affiliations:** 1 V.I. Il’ichev Pacific Oceanological Institute, Far East Branch, Russian Academy of Sciences, Vladivostok, 690041 Russia; 2 JASCO Applied Sciences Ltd, Dartmouth, NS B2Y 4S3 Canada; 3 ExxonMobil Exploration Company, Spring, TX 77389 USA; 4 JASCO Applied Sciences Ltd, Victoria, BC V8Z 7X8 Canada; 5 Lucas, TX 75002 USA

**Keywords:** Russia, Sakhalin Island, Real-time acoustic telemetry, Behavioral response mitigation, Gray whales

## Abstract

Exxon Neftegas Ltd. (ENL) carried out three 4D seismic surveys during the summer of 2015. Seismic operations in two of these fields (Odoptu and Chayvo) ensonified the nearshore feeding area of Korean-Okhotsk (western) gray whales (*Eschrichtius robustus*), potentially disturbing feeding activities. Following model-based optimization of the source design to minimize its lateral acoustic footprint, pre-season modeling was used to compute the acoustic exposure along each survey line. Real-time acoustic data facilitated implementation of mitigation measures aimed to minimize disturbance of whales. Acoustic data originated from underwater recorders deployed on the seafloor. Two complementary approaches were used to transmit recorded sound data to a computer housed at the Central Post (CP), where decisions regarding mitigation shut downs were made. In the first approach, a limited bandwidth (2–2000 Hz) sampling of the data was transmitted via cable to a surface buoy, which relayed these data to a shore station up to 15 km away via digital VHF telemetry. At the shore station, acoustic impulses from the seismic surveys were processed to compute impulse characteristics in the form of estimates of sound exposure level and peak sound pressure level, as well as one-minute-average 1/3-octave power spectral density coefficients, which were then transmitted to the CP via the internet. In the second, the pulse characteristics were computed through algorithms running on an onboard processor in each recorder’s surface buoy and sent directly to the CP computer via an Iridium satellite uplink. Both methods of data transfer proved viable, but Iridium transmission achieved the goal without the need for any shore based relay stations and is therefore more operationally efficient than VHF transmission. At the CP, analysts used the real-time acoustic data to calibrate and adjust the output of pre-season acoustical model runs. The acoustic footprint for the active seismic source, advancing synchronously with the motion of the seismic vessel and changing as the sound propagation environment changed, was computed from the calibrated and adjusted model output and integrated through the software Pythagoras with locations of gray whales provided by shore-based observers. This enabled analysts to require air gun array shutdowns before whales were exposed to mean square sound pressure levels greater than the behavioral response threshold of 163 dB re 1 μPa^2^. The method described here provides a realistic means of mitigating the possible effects of air guns at a behavioral response level, whereas most seismic surveys rely on pre-established mitigation radii to manage the risk of injury to a whale.

## Introduction

**Fig. 1:**
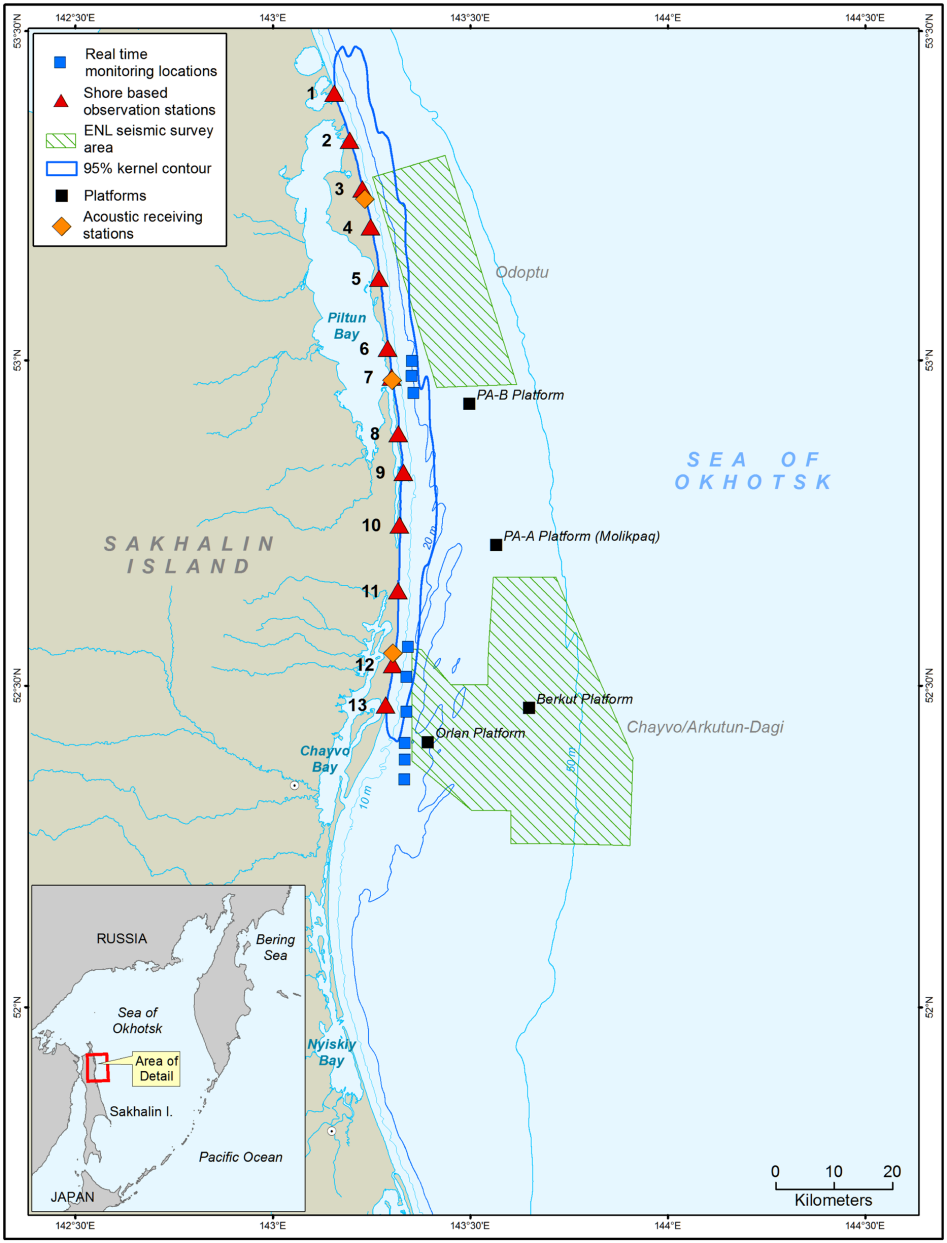
Map of the area showing the real-time monitoring locations where telemetric acoustic recorders were deployed and the shore based monitoring stations

The shallow water region of the northeast Sakhalin shelf, starting south of the mouth of Piltun Bay and extending northwards up the Sakhalin coast, is the most important known summer and fall feeding area for the Korean-Okhotsk (western) gray whale (*Eschrichtius robustus*) population. This nearshore feeding area is generally in water depths of less than 20 m, and gray whales are present, predominantly, within approximately 1.5 km of the shore (Gailey, 2013; Gailey et al., 2016; Muir et al., 2015, 2016). A second feeding area (the offshore feeding area) was discovered in 2001 in deeper water (30–60 m) approximately 20 km southeast of the mouth of Chayvo Bay. Outlines of the nearshore feeding area, based on the 95% kernel density contour, are shown in Fig. 1. The Korean-Okhotsk (western) gray whale population is listed as “Category 1” status in the Red Book of Russia and, at the time of the seismic survey, as “Critically Endangered” by the International Union for the Conservation of Nature (Cooke et al., 2018). Based on a recent re-evaluation, including not only gray whales observed off Sakhalin but also off Kamchatka, the status of the western gray whale on the IUCN Red List has been changed to ‘Endangered’ (Cooke et al., 2018).

The Sakhalin-1 license area fields Odoptu and Chayvo/Arkutun-Dagi are located near the gray whale feeding areas. In the summer of 2015, Exxon Neftegas Limited (ENL) conducted 4D seismic surveys in these three license areas using three seismic vessels. Disturbance of gray whale feeding activity due to exposure to air gun sounds was of great concern, especially in the nearshore feeding area adjacent to Odoptu and Chayvo, the only location near Sakhalin where mother-calf pairs have been observed (Tyurneva et al., 2017).

The key mitigation approach to minimizing disturbance during operations required shut down of air guns before gray whales in the nearshore feeding area were exposed to mean square sound pressure levels (SPL) above 163 dB re 1 μPa^2^,^1^ which previous studies suggested was the level at which 10% of gray whales were observed to stop feeding (Malme et al., 1988). Although source levels from the air gun array were constant throughout the surveys (that is, the same air gun array was used in all surveys), the sound propagation environment changed in both time and space. With that in mind, a single static model of an acoustic footprint would not provide a realistic representation of what sound exposure whales swimming in the feeding area might receive.

ENL developed a mitigation plan that relied on several steps: (1) optimization of the array design to minimize its acoustic footprint; (2) pre-season modeling of acoustic footprints of the air gun array at representative locations in the project area, (3) recording and subsequent real-time transmission of acoustic data during operations to a central location, known as the Central Post (CP), during surveys, (4) use of the real-time data to calibrate and adjust acoustic footprint models, and (5) overlaying of the calibrated and adjusted acoustic footprints with whale locations provided by shore-based observers. Ultimately, the combined acoustic footprint and whale location data were used to determine when air gun shut down procedures had to be implemented to avoid exposing whales to SPLs above 163 dB re 1 μPa^2^ (Aerts et al., 2022).

The complex hydrodynamic conditions present on the northeast Sakhalin shelf, such as a tidal currents of up to 1.5 m/s and the shallow 10–20 m water depths, necessitated the development of specialized acoustic recorders. These recorders were optimized for the strong flow noise caused by tidal currents and water particle motion at the bottom in surface storm waves and swells. Furthermore, the spatial and temporal extent of the 2015 seismic surveys required real-time acoustic transmitting capabilities well beyond those previously implemented. During the 2001 Odoptu seismic survey, anchored sonobuoys were used with analog VHF-FM radio telemetry links, which had extremely limited dynamic range and very short operational times (Rutenko et al., 2007). Following the 2001 survey the design moved to digital technology, and over time resulted in the development of Autonomous Underwater Acoustic Recorders (AUARs) with greater dynamic range and optional radio and Iridium telemetry functions (RI-AUARs) (Rutenko et al., 2015) capable of transmitting data over a large area for an extended duration, thanks in part to longer battery life.

This paper describes the newly designed acoustic monitoring equipment, the real-time data transfer and processing mechanism, and the use of measured levels to calibrate the pre-survey model estimates of the 163 dB re 1 μPa^2^ seismic impulse SPL isopleth, which was integrated on an operational display with the locations of gray whales received from behavioral observers at onshore stations. The work also assesses the usefulness of VHF data transmission relative to Iridium data transmission.

## Pre-season modeling

### Optimal array configuration

Sound levels produced during seismic surveys were minimized through air gun array design prior to field operations. This required investigation of various seismic array configurations to reduce the size of the 163 dB re 1 μPa^2^ SPL isopleth while maintaining the quality of seismic imaging. Because the seismic lines were to be acquired parallel to the coast, the broadside (perpendicular to the vessel track) shoreward lobe of the array acoustic emission footprint was most critical to the ensonification of the nearshore feeding area, and optimization efforts focused especially on its reduction. The process started with a 3090 in^3^ array with three strings of air guns that had been planned for an earlier survey in 2001 and was subsequently reduced in the field to a 1640 in^3^ three-string subset to decrease the 163 dB re 1 μPa^2^ SPL isopleth from an observed range of 7 km in the broadside direction to a range of 3.9 km (Rutenko et al., 2007). Against these original configurations, four new array designs were evaluated, which had varying numbers and arrangements of air guns for total volumes of 2340, 2670, 2580, and 2400 in^3^; all were two-string arrays with the exception of the 2670 in^3^ array, which was a three-string array.

The Airgun Array Source Model (AASM; MacGillivray, 2006) was used to predict the pressure signatures and directional source levels of the air gun arrays. The sound exposure level (SEL) acoustic fields around the source were modeled from the computed signatures and source levels with the Parabolic Equation (PE) based Marine Operations Noise Model (MONM), described in Austin and Chapman (2011). MONM is based on the widely accepted code RAM (Collins, 1993), modified to account for shear wave losses at the seafloor by applying a complex multiplicative factor to the seabed density (Zhang & Tindle, 1995). This approach is substantially faster than code that treats shear wave propagation in a robust sense, yet it produces results that are nearly identical to the reference approach for uniform low shear-speed, shallow-water environments with silt and sand bottoms (Hannay & Racca, 2005). The PE code does not model potential interface waves near the seafloor at frequencies of a few hertz. For per-pulse SEL estimation the propagation loss was modeled at the center frequency of each 1/3-octave band between 10 and 2000 Hz; the received sound levels were then summed over frequency to provide broadband (10–2000 Hz) exposure estimates. A Full-Waveform Range-dependent Acoustic Model (FWRAM) was used to model pressure waveforms that allowed direct calculations of peak (PK) and SPL acoustic fields. For the pulse SPL and PK metrics, the modeling was performed in 1 Hz increments and the results summed in a Fourier synthesis to yield the received wave shape from which the metrics were computed.

**Fig. 2:**
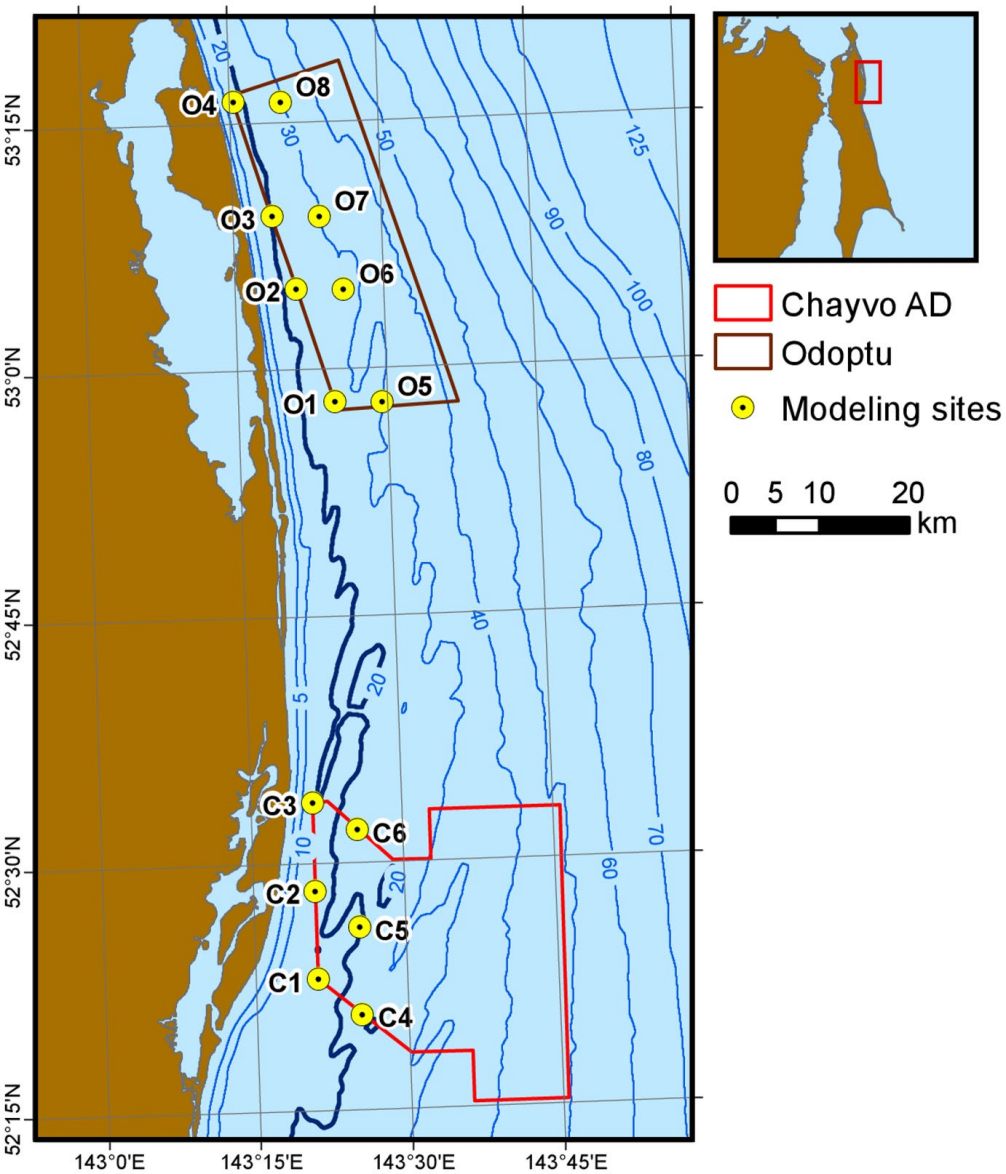
Locations offshore Sakhalin Island where the air gun array 163 dB re 1 μPa^2^ SPL isopleth was modeled for different arrays. Blue contours indicate water depth in meters

The extent of the 163 dB re 1 μPa^2^ isopleth for the different arrays was modeled at 14 locations along the northwest coast of Sakhalin Island, with eight of the sites located in the Odoptu seismic survey area and six of the sites in the Chayvo seismic survey area (Fig. 2). Half of the sites were distributed evenly along the western limit of each seismic survey (closest to shore). The others were placed 5 km to the east, following an orientation approximately perpendicular to the 20 m isobath.

The 2400 in^3^, two-string air gun array, configuration was selected as optimal for the 2015 seismic surveys from a combined standpoint of reduction of the extent of the 163 dB re 1 μPa^2^ footprint, operational efficiency, and seismic imaging quality. This array was used in the field for all the ENL seismic surveys, and all pre-season acoustic footprint modeling was based on this configuration.

### Modeling the acoustic footprint of the seismic survey lines

After selecting the air gun array configuration to be used for the seismic surveys, acoustic sound fields for the Odoptu and Chayvo operations were pre-calculated using mathematical computer models. As done previously for the assessment of the optimal source configuration, the numerical sound propagation models MONM and FWRAM were coupled with the source model AASM to compute underwater sound levels as a function of range, depth, and direction from the air gun array. The PE propagation code provided frequency-dependent sound transmission loss (TL) along a fan of radials between a source position and a 3-dimensional grid of receiver positions. When combined with directional, frequency-resolved, source levels from AASM, the result was a per pulse grid of received underwater sound levels used to estimate sound exposure within the gray whale nearshore feeding area.

Accurate model estimates were dependent on an appropriate selection of the input variables that parameterize the acoustic properties of the environment. These parameters included the water column sound speed (a depth-dependent function of temperature and salinity) and geoacoustic properties of the seafloor (sound speed and attenuation for both shear and compressional waves, and density). An average of the hydrological sampling of the water column collected over several years provided a base condition for the early summer period, when the surveys would be conducted, which exhibited a downward refracting water column sound speed profile. In the absence of specific measurements of geoacoustic properties, a base set of values for sediment density and compressional and shear wave attenuation was obtained from published information for generic sediment types (Hamilton, 1976, 1980). Sediment density (1772 kg m^–3^) and compressional wave attenuation (0.14 dB λ^–1^) were based on values for sandy silt on the continental terrace for terrigenous sediments. Shear wave attenuation (13.6 dB λ^–1^) was based on the average of values for diluvial sand and clay (19.8 dB λ^–1^) and for diluvial sand (7.4 dB λ^–1^). The approach for selecting compressional and shear wave speeds considered a finite set of possible combinations of these parameters to maximize agreement of predicted TL with measurements from dedicated sound propagation studies conducted over several source-receiver paths in the region. The possible values included two compressional speeds (1750 m s^–1^ and 2000 m s^–1^) and three shear speeds (100 m s^–1^, 200 m s^–1^, and 300 m s^–1^). The fit against data (Hannay & Racca, 2005) was accomplished by choosing the set of parameters that minimized the mean difference between model and data in 1/3-octave bands over the 50 to 500 Hz frequency range. The resulting parameters, slightly adjusted through later matching of model results with sound level measurements for offshore industrial activities in the same region, defined the model case for a representative set of propagation conditions (Tables 1 and 2).

**Table 1:** Estimated elastic parameters for the propagation model

Depth below sea floor (m)	P-wave speed (m s^–1^)	P-wave attenuation (dB λ^–1^)	S-wave speed (m s^–1^)	S-wave attenuation (dB λ^–1^)	Density (kg m^–3^)
0	1652	0.14	150	13.6	1772
500	2152	0.14			1772
> 500	2152	0.14			1772

**Table 2:** Estimated sound speed parameters for the propagation model

Depth (m)	**0**	**0.9**	**2.5**	**3.1**	**5.1**	**6.8**	**8**	**9**	**10.2**	**11.5**	**30.0 + **
Sound speed in water (m s^–1^)	1469	1469	1467	1466	1461	1456	1452	1448	1446	1444	1444

Two types of model outputs were generated. To fulfill the requirement of real-time model calibration, SEL values at the locations of the RI-AUARs were computed for every 10th planned source point (187.5 m) along the seismic survey lines. The levels were calculated for a receiver depth near the seafloor corresponding to the AUAR hydrophone placement. For this modeling only SEL quantities were estimated, and these were indexed by source position along each survey line to be compared in the field to real-time SEL measurements telemetered from the AUARs. The second model output consisted of planar grids of SPL computed for every 10th source point from the pre-plots. The SPL field grids were calculated through a multi-step process of estimation of per-pulse SEL and conversion to SPL due to the fact that direct estimation of SPL using FWRAM over the whole grid, while possible in principle, would have been prohibitively intensive computationally if performed for every source position and direction of propagation.

**Fig. 3:**
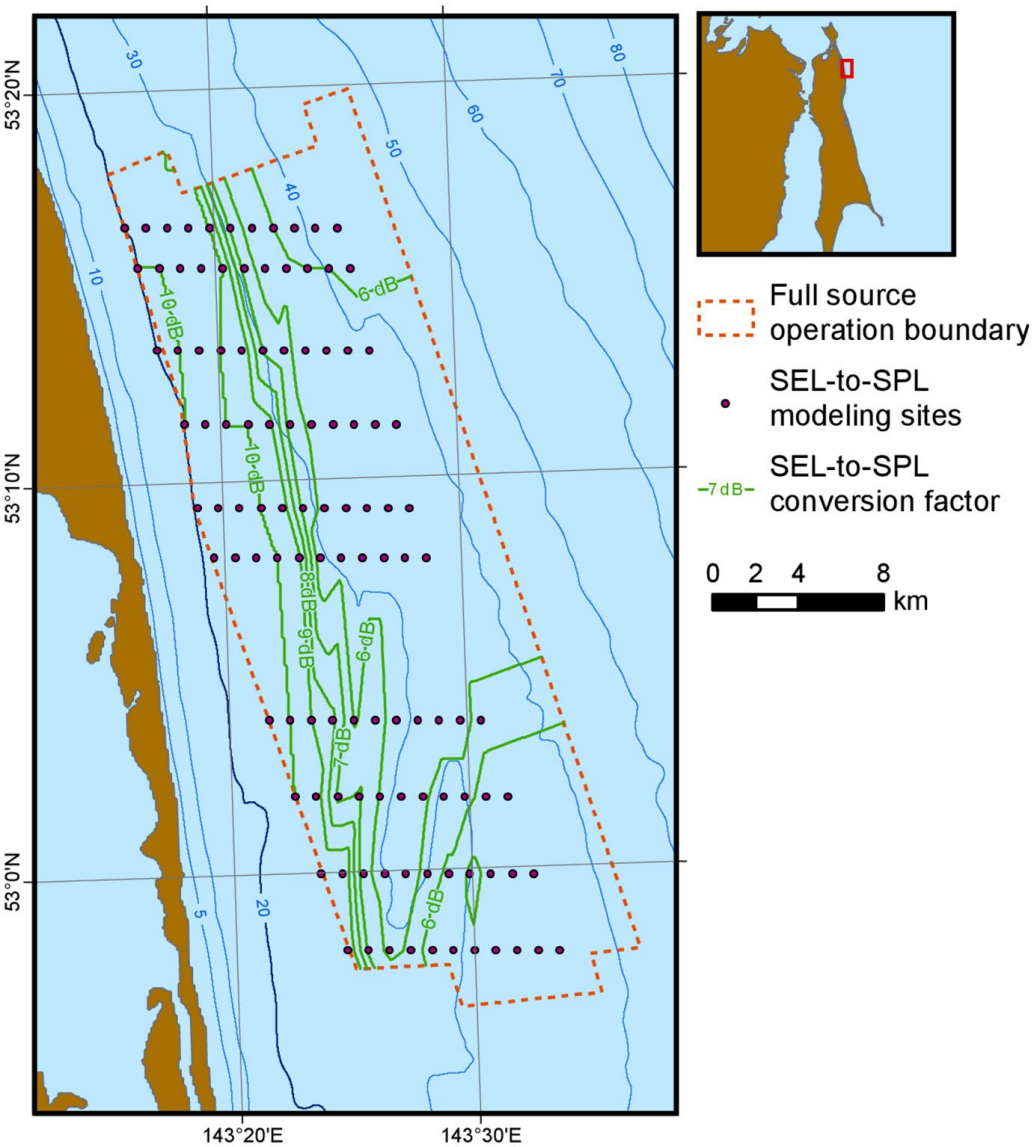
Source modeling locations along ten transects used to generate the SEL to SPL conversion factor grid (shown as contours) for the Odoptu area

The sequence of steps was as follows:

1. A planar grid of per-pulse SEL estimates was obtained for each modeled source position by running MONM for a full set of radials (only on the shoreward side of each acquisition line) and taking the maximum over depth to yield the most conservative value at each grid point.
2. At ten transects for Odoptu (Fig. 3) and eight for Chayvo, each transect containing 11 points with a 1 km shift in X coordinates, FWRAM modeling was performed along the shoreward broadside radial relative to the towing azimuth of the array. The modeling generated both SPL and SEL values, and again the maximum over depth of each was taken to generate a planar grid.
3. A set of SPL-SEL differences was computed over the FWRAM-generated shoreward broadside radials at locations where the estimated SPL was in the 162–164 dB re 1 μPa^2^ range. The average of the differences for this selected subset was taken as the conversion factor for that location. The factor was capped at 10 dB to avoid runaway outliers.
4. A conversion factor grid was compiled based on the values at modeled locations. The grid was used to estimate the SEL-to-SPL conversion factor at any modeled location within the grid boundaries and convert SEL values obtained from MONM modeling to SPL values.

## Monitoring air gun sounds

### Acoustic monitoring locations

ENL used three seismic vessels to conduct 4D seismic surveys in the Odoptu and Chayvo/Arkutun-Dagi fields during the summer and autumn of 2015*.* Each of these vessels towed two 2400 in^3^ air gun arrays at a depth of 5 m. Sounds generated by the seismic survey activities were logged by 40 Autonomous Underwater Acoustic Recorders (AUARs) at 48 locations (Rutenko et al., 2022). The recorders intended for use in the real-time monitoring were equipped with VHF-Iridium surface buoys (RI-AUARs; Rutenko et al., 2015) and deployed along the 20 m contour nearshore of the Odoptu and Chayvo seismic survey areas (Fig. 1). These buoys transmitted dual data streams, one using VHF signals and the other via an Iridium satellite uplink.

The RI-AUARs monitored and characterized the low-frequency acoustic impulses generated by the seismic surveys and propagating into the nearshore feeding area. As many as three RI-AUARs were deployed in Odoptu and six in Chayvo over the course of the seismic surveys.

### Equipment specifications and improvements

A new generation of high-performance low-power microcontrollers with the ability to stream digitized data directly to solid-state storage devices (SD cards) was used for the 2015 real-time acoustic monitoring recorders. Avoiding the use of general-purpose single board computers and hard disk drives in the AUARs led to a substantial decrease (by a factor of more than 15) in power consumption. Additionally, the use of an Iridium satellite radio telemetry link not only removed any transmission range constraints but also enabled operational control of the recorders. The new RI-AUAR is capable of recording acoustic pressure with a bandwidth of 2–15,000 Hz and a dynamic range of 145 dB, transmitting acoustic data with a bandwidth of 2–2000 Hz via a 16-bit digital VHF radio link over distances of up to 15 km, performing a preliminary pre-processing (0.5 s windows) of the acoustic data recorded with a bandwidth of 2–2000 Hz (24-bit data) using a controller in the surface buoy, and transmitting a 270-byte message via an Iridium satellite link at 60 s intervals to an onshore receiving station. The message contained the following data: date, time, 120 PK values, 120 SEL values, and 22 60-s average 1/3-octave power spectral density coefficients.

### Real-time data transmission

**Fig. 4:**
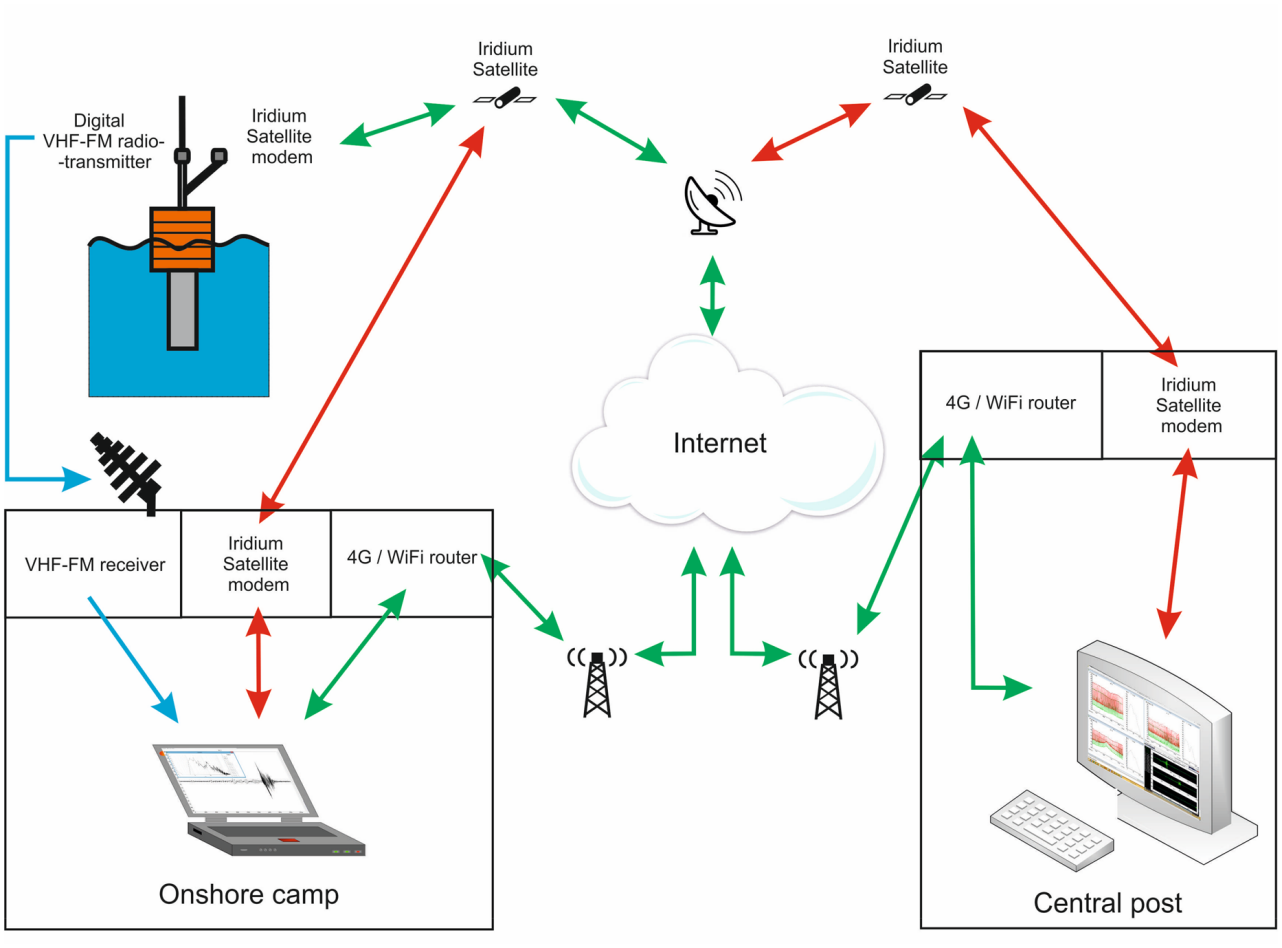
Schematic showing the data transmission pathways from the RI-AUARs to the Central Post (CP)

The acoustic data were transmitted from the RI-AUAR antenna on the surface float to the Central Post (CP) via two pathways (Fig. 4), as previously noted, one relying on VHF transmission and the internet and one relying on an Iridium uplink and the internet. This was done primarily to validate the new technology of onboard processing and Iridium telemetry against the proven, but range limited, VHF telemetry with the goal of eventually phasing out the latter.

VHF signals were transmitted to onshore camps where the digitally encoded acoustic data (bandwidth 2–2000 Hz) were processed by a program that automatically identified and characterized the acoustic impulses in 60-s intervals (Gritsenko, 2013). The software first performed bias removal by median averaging and calculated the average values of sound pressure at intervals of 30 ms. Impulses were identified if the acoustic envelope exceeded the 98th percentile value. The time interval that encompassed the pulse started 1 s before the identified time and ended 3 s past it. The characteristics of each impulse were calculated for a time window that contained 90% of the energy of the impulse. Values of SEL, PK, and 1/3-octave spectral values were estimated for each pulse. Finally, the results of these analyses were transmitted to the CP via the internet.

In parallel, the acoustic data were autonomously analyzed by a computer in the RI-AUAR surface float, and the results of the analyses were sent to the CP via the Iridium channel in 270-byte packets at 60 s intervals. Each packet contained 120 30 s SEL values, 120 PK values, and 22 60-s average 1/3-octave spectrum coefficients, as well as the date and time.

**Fig. 5:**
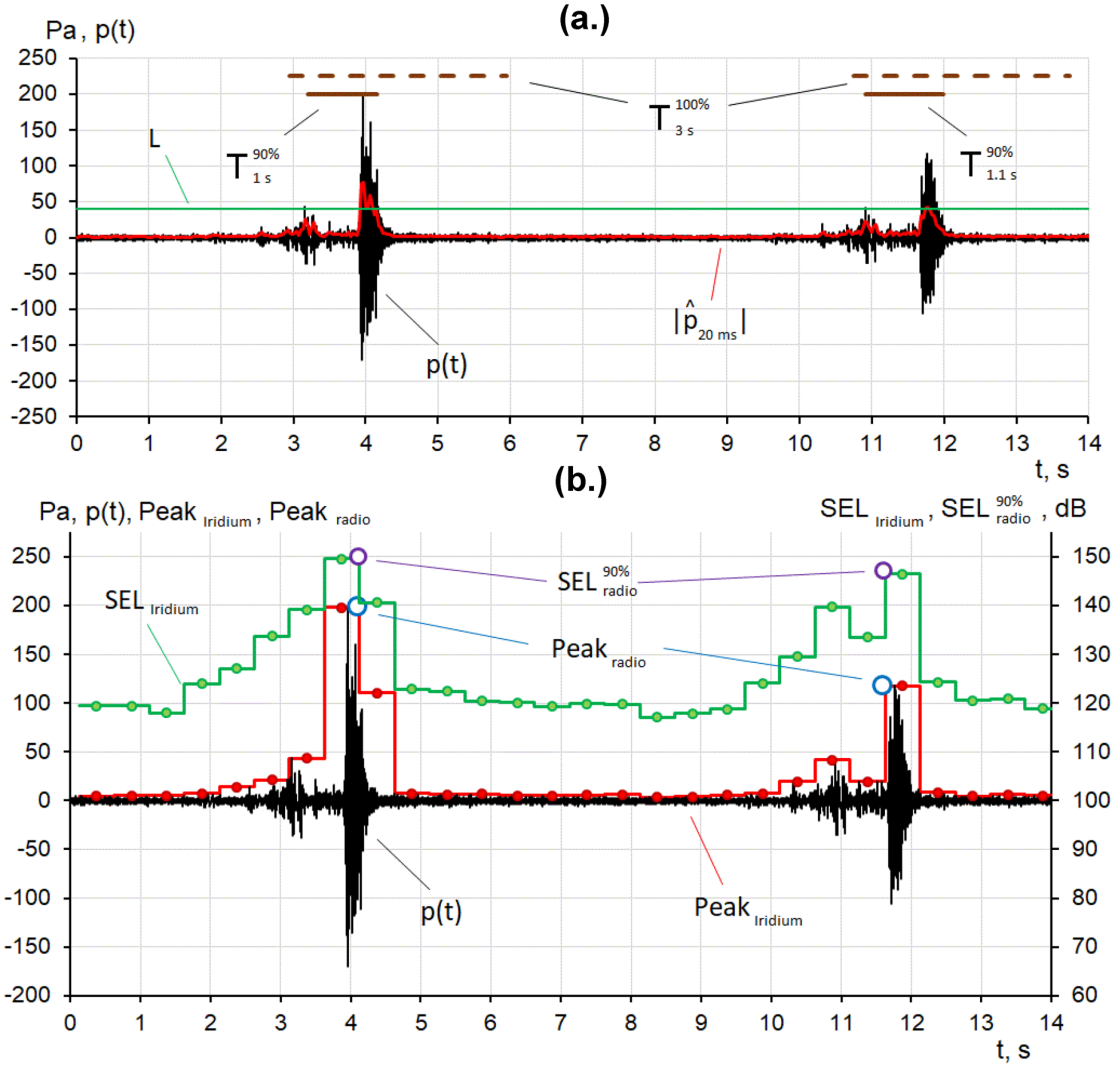
Comparison of the analysis of the characteristics of acoustic impulses computed **a** at the acoustic reception camp from data transmitted via VHF, and **b** at the RI-AUAR surface buoy and transmitted via Iridium

The impulse characteristics estimated from data transmitted via VHF and processed at onshore camps largely matched the impulse characteristics calculated at the RI-AUAR and transmitted via Iridium (Fig. 5). The Iridium link was also used to control the operation of each RI-AUAR. The operator could turn the VHF and Iridium transmission on and off and control the output level of the VHF link. A backup system for the transmission of data and commands, in which Iridium transceivers were used to relay the data, started automatically in the event of problems with the default Internet framework.

**Fig. 6:**
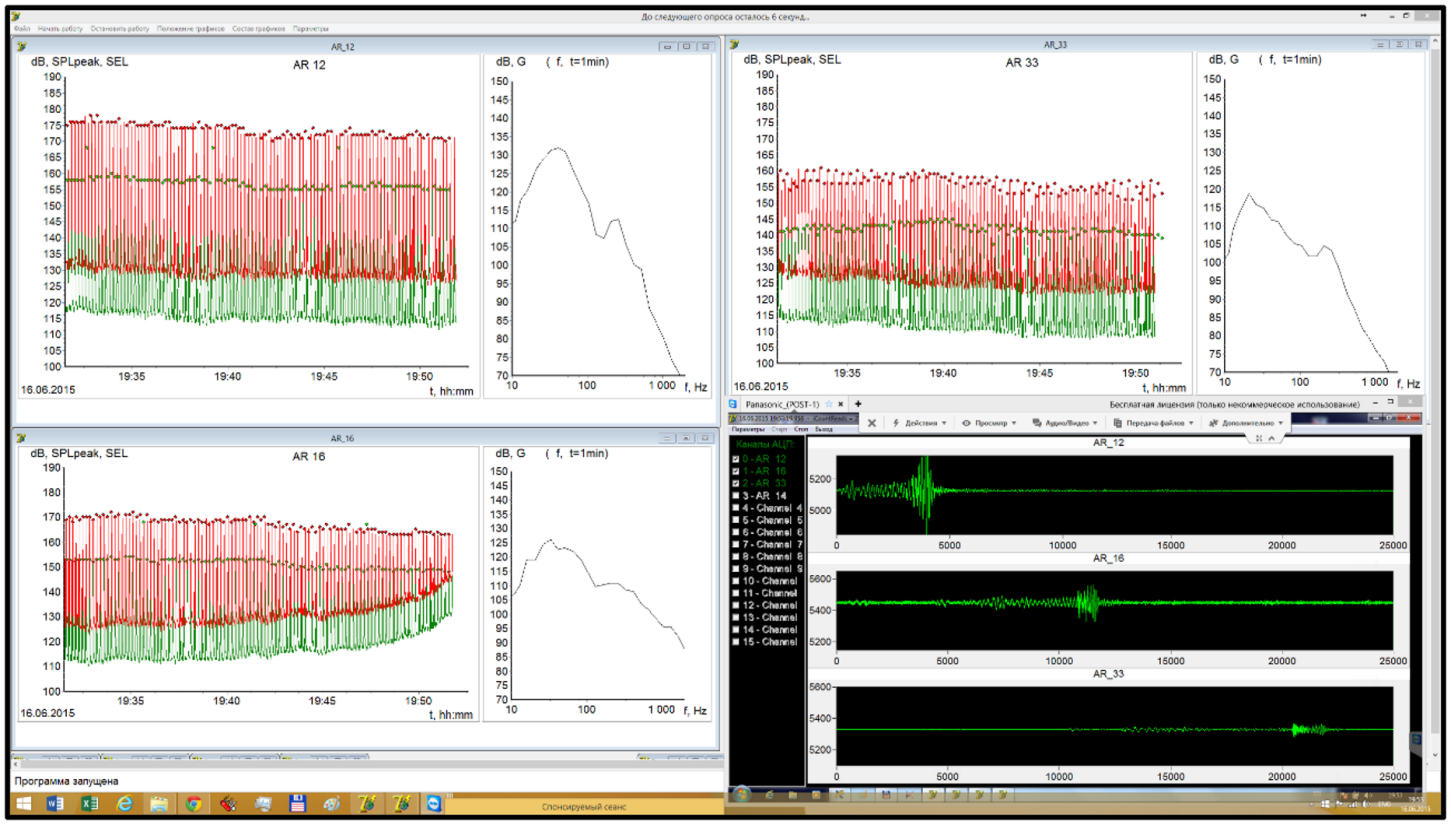
Screen shot of the display program at the CP. For each monitored RI-AUAR, the PK and SEL values transmitted via VHF and Iridium and the time domain plots (via VHF) are displayed

All transmitted data were displayed on the CP computers (Fig. 6). Each window corresponds to the output from an RI-AUAR; the plot parameters of the windows can be adjusted independently. The PK and SEL values estimated at the RI-AUAR and transmitted via Iridium are plotted as points, while the equivalent impulse characteristics generated onshore and transmitted via the internet are plotted as lines.

## Calibration of pre-season acoustic footprints

**Fig. 7:**
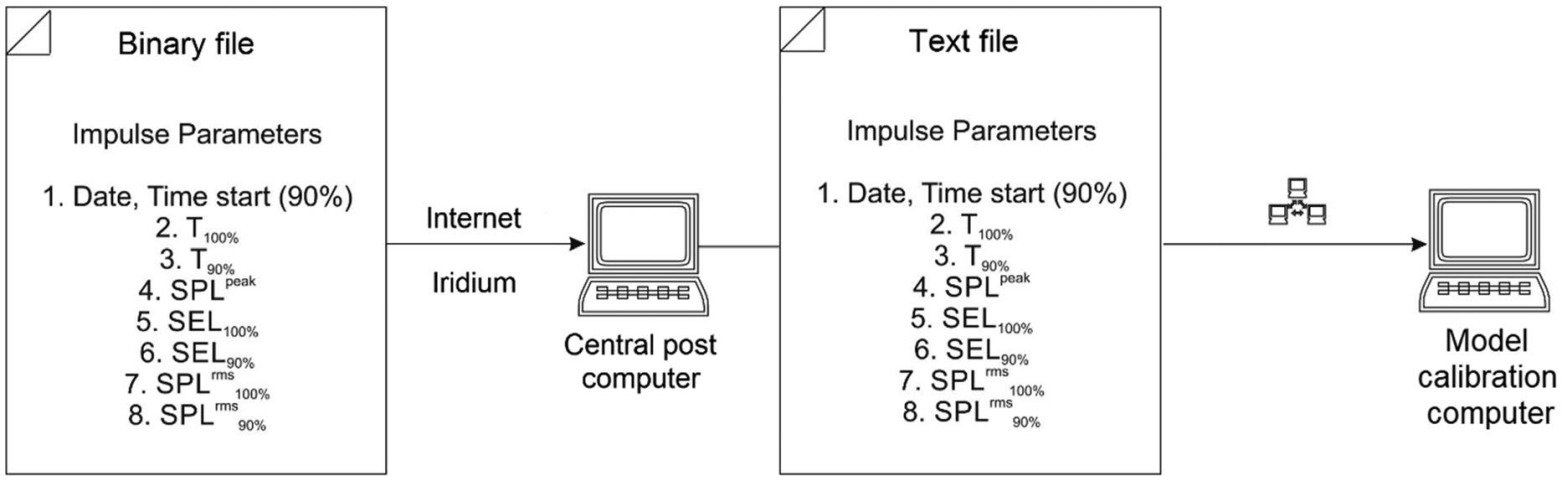
Schematic showing the real-time data flows of the estimated characteristics of recorded impulses to the calibration computer for comparison to the modeling results

The transmission of the impulse characteristics to the CP was the first stage in the real-time acoustic monitoring process. The impulse characteristics transmitted from each RI-AUAR were aggregated and saved in a text file every minute. The impulse characteristic files were shared with the model calibration computer via the local network (Fig. 7). This calibration computer was used to compare the estimated characteristics of the acoustic pulses to pre-survey computer modeling.

**Fig. 8:**
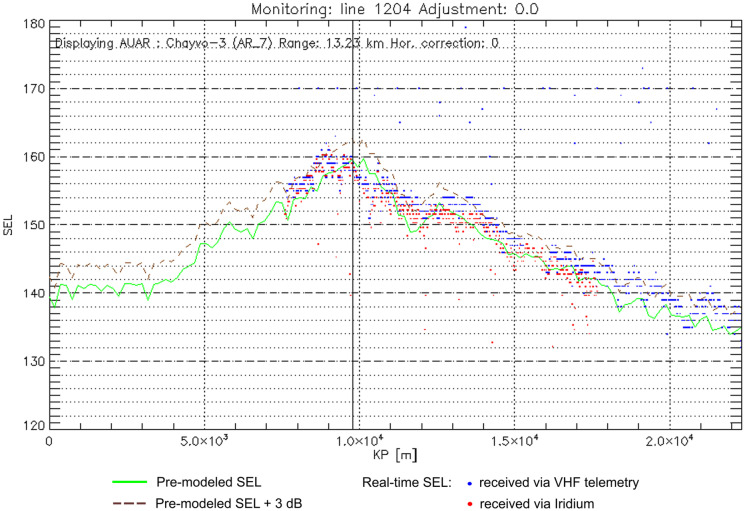
Example of the calibration graph displayed on the operator screen for acquisition of Chayvo Line 1204 with real-time data from monitoring point Chayvo-3. Solid green line – pre-modelled SEL; dashed brown line – 3 dB buffer over pre-modelled SEL; blue and red dots – real-time SEL readings from AUAR received via VHF telemetry and Iridium channels, respectively

The per-pulse received level (SEL) sequences from the RI-AUARs were used in real-time during the seismic surveys to calibrate and adjust model-based estimates of received levels as follows. As the source vessel progressed, the received pulse levels at a given station were plotted as a function of vessel progress or source point number using a custom software application that also overlaid a graph of the corresponding modeled levels to enable comparison. The comparison software tracked real-time data streams from all available monitoring stations allowing selection of the specific station of interest for graphical display. Figure 8 shows an example of the operator screen that allowed comparison between the pre-modeled and real-time monitoring data along a seismic survey line. Based on the visual comparison between the measured and modeled pulse level traces, a decibel offset was selected if required to adjust the sound level prediction. In parallel to this process, separate software tracked the active seismic source vessel position and at regular time intervals (30 s) generated the current 163 dB re 1 μPa^2^ SPL isopleth contour as a GIS shape file that was transmitted to the operational data aggregation and display system. The contour was generated based on the nearest available pre-modeled SPL grid with application of the aforementioned adjustment, if required.

**Fig. 9:**
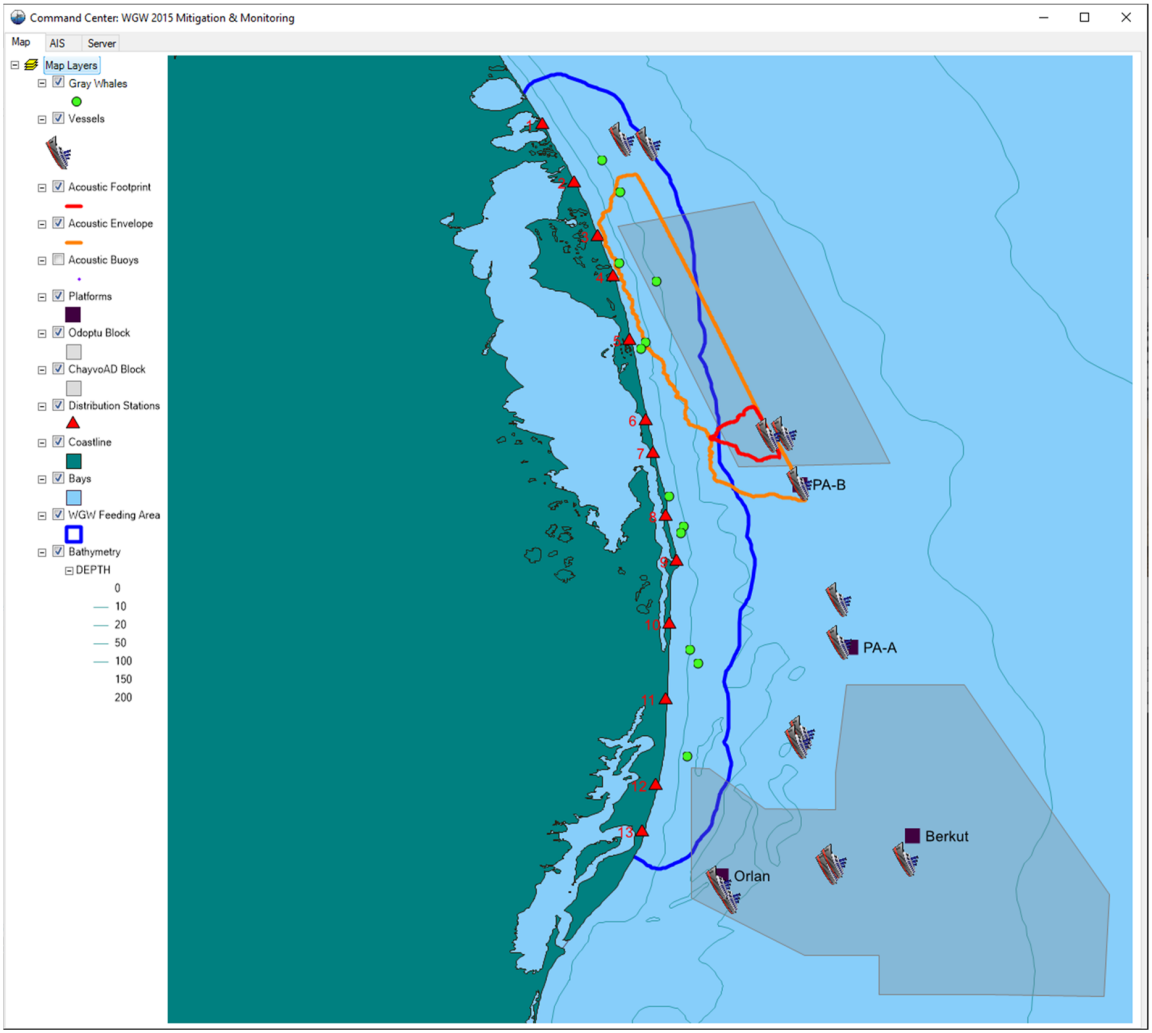
Information display generated with Pythagoras™ software showing AIS vessel information, real time gray whale locations (colored dots), shore-based whale tracking stations (red triangles), outline of nearshore feeding area (blue contour), and 163 dB re 1 μPa^**2**^ SPL footprint for the active seismic vessel (instantaneous: red contour; full-line envelope: orange contour)

The Pythagoras system was used at the CP to visualize—in real time and on a single map display—the 163 dB re 1 μPa^2^ SPL isopleth and the locations of gray whale sightings, along with real-time vessel movements from AIS (Fig. 9). This system allowed personnel based in the CP to follow the acoustic footprint and its overlap with the 95% density contour of the nearshore feeding area as the vessel was moving along the line for the Odoptu and Chayvo seismic surveys. Both the per-pulse footprint and the acoustic envelope of the entire line were displayed. Based on this information, shut down and restart instructions were communicated directly to the seismic survey vessels.

## Conclusions

During the summer of 2015, ten autonomous underwater acoustic recorders with VHF and Iridium telemetry systems were used to provide real-time acoustic data for mitigation purposes. The real-time Iridium system performed well, and the data transmitted to the CP were comparable to the data transmitted via the VHF channel. The operational complexity involved with the VHF transmission path was much greater than the Iridium transmission path. During future surveys where similar data transfer capabilities will be required, broadband Iridium alone will be used for real-time data transmission.

The real-time acoustic monitoring allowed model calibration and adjustment, which in turn provided a calibrated real-time acoustic footprint that was used for mitigation. This footprint, used in concert with whale locations provided by onshore observers, allowed analysts in the CP to instruct the vessel to implement air gun array shutdowns before whales could be exposed to sounds greater than 163 dB re 1 μPa^2^ SPL. During the operations described in this paper, four behavioral shutdowns were implemented due to gray whales approaching the 163 dB re 1 μPa^2^ SPL footprint (Aerts et al., 2022).

The method described here provides a realistic means of mitigating the possible effects of air guns at a behavioral response level, whereas most seismic surveys only rely on pre-established mitigation radii to manage the risk of injury to a whale. The larger extent and greater dependence on sound propagation conditions of the behavioral response footprint demand substantially more sophisticated modeling, monitoring, and calibration approaches than the static estimation of the safety (injury) radius used in the majority of surveys. The unique conditions present in the nearshore Sakhalin shelf both enabled and justified this more advanced mitigation strategy.

## Footnotes

1 The definition of the SPL metric as mean square pressure level with reference value 1 µPa^2^ conforms with the acoustical terminology standards ISO 18405 and ANSI S1.1, and adheres to the original definition of the decibel in terms of a logarithmic ratio of power quantities. Earlier literature may refer equivalently to root mean square (rms) pressure level with reference value 1 µPa.
